# Cytogenetic analysis of some species of *Cyphomyrmex* Mayr, 1862 and *Apterostigma* Mayr, 1865 (Formicidae, Myrmicinae) from the Guiana Shield

**DOI:** 10.3897/compcytogen.20.175870

**Published:** 2026-03-25

**Authors:** Rodrigo Batista Lod, Luísa Antônia Campos Barros, Linda Inês Silveira, Esthefanne de Araújo Leitão, Gisele Amaro Teixeira, Hilton Jeferson Alves Cardoso de Aguiar

**Affiliations:** 1 Programa de Pós-graduação em Biodiversidade Tropical, Universidade Federal do Amapá, Campus Marco Zero do Equador, Macapá, Amapá, 68.903-419, Brazil Programa de Pós-graduação em Biodiversidade Tropical, Universidade Federal do Amapá, Campus Marco Zero do Equador Macapá Brazil; 2 Universidade Federal do Amapá. – Oiapoque, n°3051, Bairro Universidade, Oiapoque, Amapá, 68980-000, Brazil Universidade Federal do Amapá. – Oiapoque Amapá Brazil; 3 Laboratório de Citogenética de Insetos, Departamento de Biologia Geral, Universidade Federal de Viçosa, Viçosa, Minas Gerais, 36570-900, Brazil Laboratório de Citogenética de Insetos, Departamento de Biologia Geral, Universidade Federal de Viçosa Viçosa Brazil

**Keywords:** Chromosome, Formicidae, Neotropical, Attina, rDNA, microsatellites

## Abstract

Ants are present in different environments and regions of the planet, showing little-known biodiversity in the Neotropics, especially among fungus-growing ants (the subtribe Attina). Recent studies seek to combine morphological analysis with other methods, such as cytogenetics, to better define and differentiate species. In this sense, cytogenetic analyses have been important for the study of ants, characterizing chromosome number and morphology, and mapping rDNA genes and microsatellites, which generate important information about the evolution and taxonomy in Attina species. In the Amazon region, there are still few studies that include cytogenetics in their research. In this study, we cytogenetically characterized five fungus-growing ants, belonging to the genera *Cyphomyrmex* Mayr, 1862 and *Apterostigma* Mayr, 1865, from the northernmost region of Amapá state within the Guiana Shield region of Brazil. The nests were captured by active search, and the larvae had their brain ganglia extracted to provide metaphase chromosomes. The karyotypes were determined using Giemsa or DAPI staining, and 18S ribosomal DNA (rDNA) and (GA)_n_ microsatellite repetitive sequences were physically mapped with FISH technique with specific probes. *Cyphomyrmex transversus* Emery, 1894 had 2n = 24 chromosomes (18m+6sm) and *Cyphomyrmex laevigatus* Weber, 1938 n = 7, all metacentrics. In both species, the rDNA clusters were restricted to a single chromosome pair. In *C. transversus* the rDNA clusters were mapped to the long arm of the larger submetacentric chromosome pair, while in *C. laevigatus* they were on the short arm of the fifth chromosome (haploid individuals). These data are aligned to *Cyphomyrmex* species previously studied from this region. Although the FISH protocol was successfully applied to *Cyphomyrmex* species, it was unable to localize rDNA sites in the chromosomes of all three *Apterostigma* species, suggesting that methodological adjustments are required for an effective application to this genus. In *Apterostigma*, the largest chromosome number of the genus was identified in *Apterostigma tropicoxa* Lattke, 1997, with 2n = 54 chromosomes, while *A. jubatum* Wheeler, 1925 and *A. andense* Lattke, 1997 had 2n = 22 and n = 11 respectively but were strikingly diverse in their karyotype configurations. In *C. laevigatus* and *C. transversus* the microsatellite (GA)_n_ clusters were scattered on all chromosomes. While *A. jubatum* also had a scattered distribution pattern of this microsatellite on all chromosomes, the other *Apterostigma* species showed more complex patterns. In *A. andense* these microsatellite sequences were more concentrated at the ends of some chromosomes, while in *Apterostigma tropicoxa* they were almost absent in the short arms of several submetacentric and subtelocentric chromosomes. The cytogenetic data for Amazonian species in this study highlight the chromosomal diversity among fungus-growing ants, particularly within the genus *Apterostigma*, providing useful insights into the karyotype evolution of these ants and paving the way for further cytogenetic research in the Amazon region.

## Introduction

Within Myrmicinae, the fungus-growing ants (Attini: Attina) are notable for maintaining a symbiotic relationship and relying exclusively on fungus cultivation for food (Schultz and Brady 2008). These ants form a monophyletic group, comprising approximately 247 species spread across 20 genera, which includes the genera *Cyphomyrmex* Mayr, 1862 and *Apterostigma* Mayr, 1865 (Hanisch et al. 2022; Bolton 2025). They are exclusively found on the American continent, primarily in tropical regions, where their biodiversity reaches its peak (Schultz and Brady 2008; Fernández et al. 2021; Hanisch et al. 2022).

Cytogenetic studies enhance the assessment of hidden biodiversity in natural habitats. Despite the high ant diversity in the Neotropics, karyological data on these insects are limited and do not cover a significant portion of the species in the region (Mariano et al. 2019). The karyotype of a species is useful for taxonomic purposes, even suggesting the establishment of new taxa such as in the case of *Amoimyrmex* Cristiano et al., 2020, but chromosomal data can also enrich the discussions over the population dynamics within the same species, leading to important conclusions about habitat fragmentation and species vulnerability (Mariano et al. 2008; Lorite and Palomeque 2010). Recent studies have highlighted the importance of cytogenetics in ant groups which taxonomical status relied only on morphological identification, and yet they have clear karyotypic differences that go beyond minor variations between different populations (Teixeira et al. 2020; Barros et al. 2021a).

*Cyphomyrmex* currently comprises 23 described species, and the genus harbors two species complexes: minutus and rimosus (Kempf 1966; Albuquerque 2014; Bolton 2025). These species complexes are distinguished by morphological traits. To date, six taxa have cytogenetic data, with chromosomal variation ranging from 2n = 14 in *Cyphomyrmex laevigatus* Weber, 1938 (Damasceno et al. 2024) to 2n = 42 in two populations of *Cyphomyrmex transversus* Emery, 1894 (Mariano et al. 2019; Cardoso and Cristiano 2021) (Suppl. material 1). The karyotype of a population of *C. rimosus* (Spinola, 1851) from the Brazilian Atlantic Forest showed a stark divergence from the karyotype previously described for this species from a population from Central America (Murakami et al. 1998; Teixeira et al. 2023) corroborating the hypothesis of a species complex within this taxon, as previously suggested by Kempf (1966) analyzing morphological traits. In addition, three highly diverse karyotypes in *C. transversus* (2n = 18, 2n = 24/n = 12, and 2n = 42) from different locations have been described (Mariano et al. 2019; Aguiar et al. 2020; Cardoso and Cristiano 2021; Teixeira et al. 2021b), suggesting the possibility of other species complex within *C. transversus*.

*Apterostigma* currently comprises 44 valid species subdivided in two distinct groups: auriculatum and pilosum (Lattke 1997; Bolton 2025). To date, seven taxa have been studied cytogenetically, showing chromosomal variation ranging from 2n = 20 to 2n = 46 in *Apterostigma* sp. and *Apterostigma* sp. pilosum complex, respectively (Fadini and Pompolo 1996; Barros et al. 2021b) (Suppl. material 1).

The Minimal Interaction Theory (Imai et al. 1994) provides a comprehensive and elegant understanding not only of the mechanisms involved in karyotype alteration in ants, but also of the very evolution of these mechanisms. This theory is based on the distribution of heterochromatin in the karyotypes of the analyzed species. The Minimal Interaction Theory proposes that if a karyotype increases in the chromosome number, the chromosomes decrease in size, due to centric fission events. Chromosomes derived from centric fission tend to be small, and the expansion of heterochromatin may serve as a mechanism to maintain their structural stability (Imai et al. 1988). Recently, molecular cytogenetic techniques have enriched these discussions. The physical mapping of repetitive DNA sequences, such as ribosomal DNA (rDNA) and microsatellites, through fluorescence *in situ* hybridization (FISH) has provided important insights into the mechanisms of karyotypic variation and evolution among different groups of ants (Micolino et al. 2020; Teixeira et al. 2021a; Damasceno et al. 2024; Silveira et al. 2024).

rDNA genes contain highly conserved coding sequences and are part of the nucleolar organizer region (NOR) (López-Flores and Garrido-Ramos 2012; Teixeira et al. 2021a; Gokhman and Kuznetsova 2024). Although highly conserved, their chromosomal distribution patterns vary across ant species (Teixeira et al. 2021a) as well as substantially throughout the insect group, among different species and, in some cases, even within the same species (Gokhman and Kuznetsova 2024). Molecular cytogenetic data for *Cyphomyrmex* include the mapping of rDNA genes for *C. transversus*, *C. rimosus*, and *C. laevigatus*. All three species have a single chromosome pair bearing the rDNA clusters (Teixeira et al. 2021b; Teixeira et al. 2023; Damasceno et al. 2024).

Microsatellites are short tandem repeats up to nine nucleotides, and they are considered useful molecular markers due to their variable lengths (López-Flores and Garrido-Ramos 2012). Their non-random distribution across coding and non-coding regions suggests important roles in gene regulation (Li et al. 2004). Microsatellite sequences, such as motif (GA)_n_, are common in some invertebrates. In ants, it is present mainly in euchromatic regions, being absent from centromeres (Teixeira et al. 2023). Physically mapped (GA)_n_ microsatellites on chromosomes are available for *C. rimosus* (Teixeira et al. 2023) and *C. transversus* (Teixeira et al. 2021b). In the genus *Apterostigma*, only chromosomes of *A. madidiense* Weber, 1938 have been studied for mapping the microsatellites (GA)_n_ (Teixeira et al. 2022). Despite the importance of cytogenetic data for understanding chromosomal evolution, and their potential to clarify taxonomic units within Attina, few data are available for the Amazon region. Both *Cyphomyrmex* and *Apterostigma* genera have species complexes recognized by taxonomic and cytogenetic approaches. Efforts to delimit taxa within species complexes enhance our understanding of biodiversity, guiding more effective resource allocation for habitat management and conservation. Therefore, the present study aimed to expand the cytogenetic data for *Cyphomyrmex* and *Apterostigma* species from the Guiana Shield region, providing new insights into their chromosomal diversity and karyotype evolution.

## Methods

Sampling was conducted in the municipality of Oiapoque, located in the eastern Amazon region of Brazil (3.832994, -51.844899). This sampling was authorized by SISBIO/ICMBio collection permit no. 89871. Adult individuals from each colony were identified by Dr. Jacques H. C. Delabie and vouchers were deposited in the myrmecological collection of the Centro de Pesquisas do Cacau (CPDC) at the Comissão Executiva do Plano da Lavoura Cacaueira (CEPLAC), in Bahia, Brazil, record #5882.

Mitotic metaphases of fungus-growing ant species were obtained from the cerebral ganglia of larvae after meconium elimination, according to the protocol described by Imai et al. (1988). The slides were stained with 4% Giemsa or DAPI (Fluoroshield with DAPI, Sigma-Aldrich). Giemsa-stained slides were analyzed and photographed using an Olympus BX53 microscope equipped with an Olympus DP73 camera, and DAPI stained slides were analyzed and photographed using an Olympus BX53F microscope equipped with epifluorescence and an Olympus XM10 camera using Olympus cellSens© Dimension 1.6 software. Chromosomes were measured and counted using Image Pro Plus®. Karyotypes were organized using Adobe Photoshop©. Chromosome classification was based on the methodology proposed by Levan et al. (1964).

Physical mapping of microsatellite (GA)_n_ and rDNA genes was performed using fluorescence *in situ* hybridization (FISH) following the protocol of Pinkel et al. (1986) with modifications described by Teixeira et al. (2021a). The probes for the 18S rDNA gene were amplified by PCR using the primers 18SF1 (5'–GTC ATA GCT TTG TCT CAA AGA–3') and 18SR1.1 (5'–CGC AAA TGA AAC TTT AAT CT–3') designed for the bee *Melipona quinquefasciata* Lepeletier, 1836 (Pereira 2006), and genomic DNA of the ant *Camponotus rufipes* (Fabricius, 1775). The probes were indirectly labeled using digoxigenin-11-dUTP (Roche Applied Science, Mannheim, Germany) during PCR reaction and the labeling signal detected with anti-digoxigenin-rhodamine. The microsatellite probe (GA)_15_ was directly labeled with Cyanine-3 (Cy3) at the 5' terminal end (Sigma, St. Louis, MO, USA). Metaphases were analyzed and photographed using an Olympus BX53F microscope equipped with epifluorescence and an Olympus XM10 camera using Olympus cellSens© Dimension 1.6 software.

## Results

**Figure 1.**
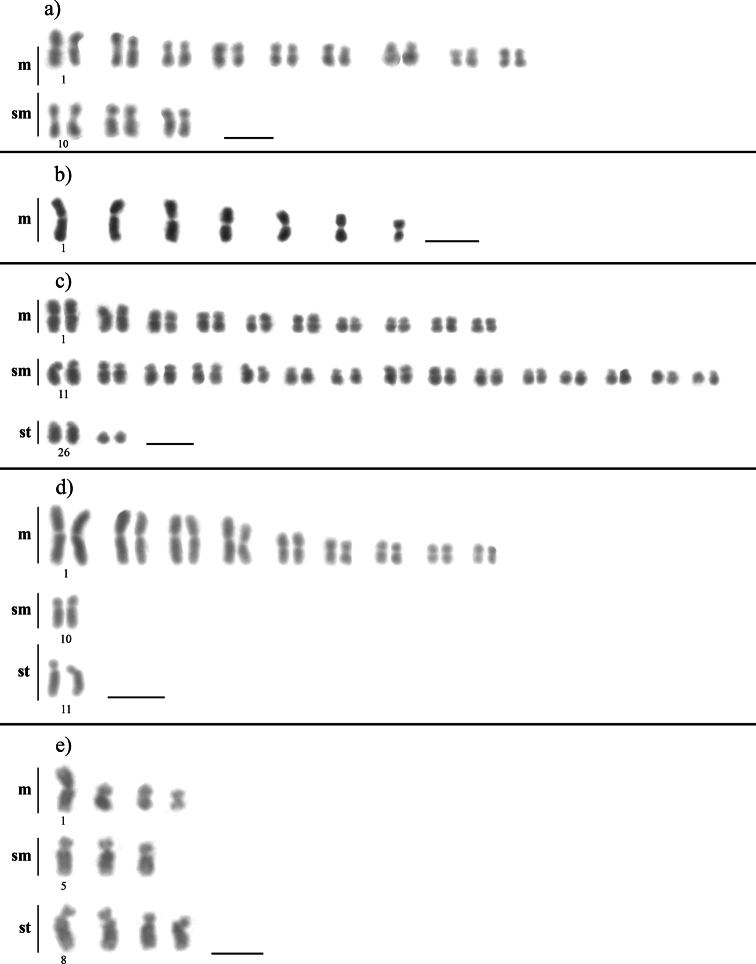
Karyotypes of the genera *Cyphomyrmex* and *Apterostigma***a***Cyphomyrmex transversus* (2n = 24) **b***Cyphomyrmex laevigatus* (n = 7) **c***Apterostigma tropicoxa* (2n = 54) **d***Apterostigma jubatum* (2n = 22), and **e***Apterostigma andense* (n = 11). Scale bars: 5 μm.

**Figure 2.**
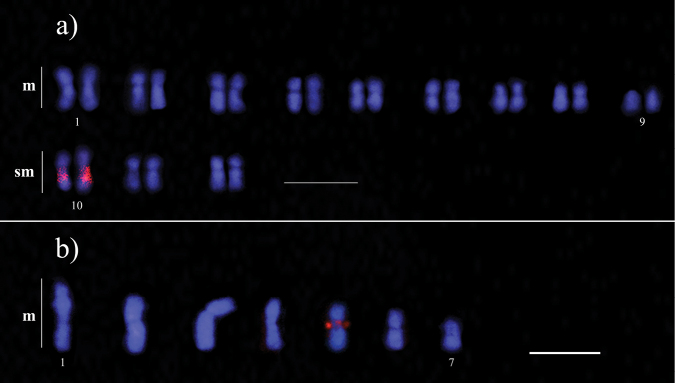
Mapping of rDNA clusters (red regions) using fluorescence *in situ* hybridization on the chromosomes of the genus *Cyphomyrmex***a***Cyphomyrmex transversus* (2n = 24), and **b***Cyphomyrmex laevigatus* (n = 7). Scale bars: 5 μm.

**Figure 3.**
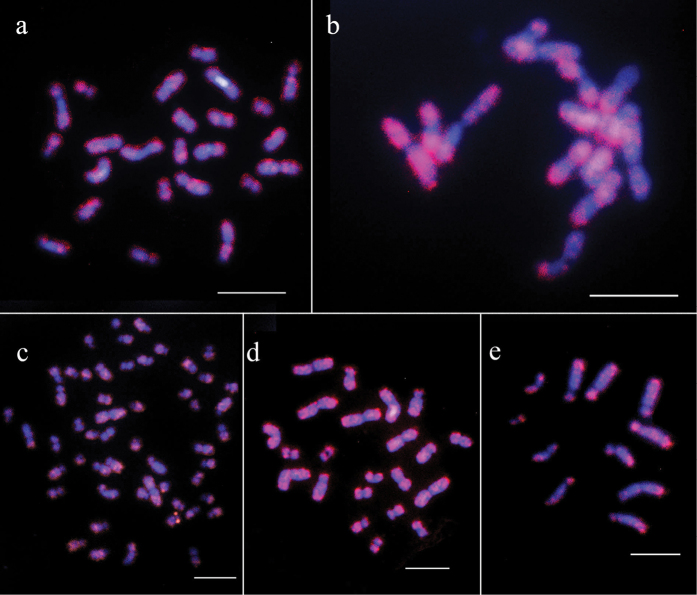
Microsatellite (GA)_n_ patterns (red regions) using fluorescence *in situ* hybridization on the chromosomes of *Cyphomyrmex* and *Apterostigma* species **a***Cyphomyrmex transversus* (2n = 24) **b***Cyphomyrmex laevigatus* (2n = 14) **c***Apterostigma tropicoxa* (2n = 54) **d***Apterostigma jubatum* (2n = 22), and **e***Apterostigma andense* (n = 11). Scale bars: 5 μm.

The chromosome number and morphology of two *Cyphomyrmex* species and three *Apterostigma* species were characterized (Table 1). Individuals belonging to three colonies of *Cyphomyrmex transversus* (Fig. 1a) presented 2n = 24 (18m+6sm). Specimens from a colony of *Cyphomyrmex laevigatus* showed a haploid karyotype with n = 7, all metacentrics (Fig. 1b). 18S rDNA probe was mapped to a single chromosome pair in the diploid karyotype (or one chromosome in the haploid karyotype) in all *Cyphomyrmex* species (Fig. 2). 18S rDNA probe was detected in the pericentromeric region of the first submetacentric pair in *Cyphomyrmex transversus* (Fig. 2a) and in the short arm of the fifth chromosome in the haploid set of *Cyphomyrmex laevigatus* (Fig. 2b). The microsatellite (GA)_n_ was found to be dispersed among both chromosome arms of the two *Cyphomyrmex* species (Fig. 3a, b).

**Table 1.** Species of the genera *Cyphomyrmex* and *Apterostigma* collected in Oiapoque, Amapá State, Brazil and their cytogenetic results. p = short chromosome arm, q = long chromosome arm. disp = dispersed chromosomal distribution. t = telomeric region.

**Species**	**2n/(n)**	**Col/Ind**	**Karyotypic formula 2n/(n)**	**rDNA**	**(GA)_n_**
*Cyphomyrmex transversus*	24	3/14	18m+6sm	Pericentromeric	disp
*Cyphomyrmex laevigatus*	(7)	1/5	14m/(7m)	Pericentromeric	disp
*Apterostigma tropicoxa*	54	1/3	20m+30sm+4st	–	disp+ q
*Apterostigma jubatum*	22	1/5	18m+2sm+2st	–	disp
*Apterostigma andense*	(11)	1/5	(4m+3sm+4st)	–	disp, t

–: *Apterostigma* species did not present 18S rDNA probe signals with the methodology used.

Samples from three colonies of species belonging to the genus *Apterostigma* were collected. *Apterostigma tropicoxa* Lattke, 1997 had 2n = 54 (20m+30sm+4st), *Apterostigma jubatum* Wheeler, 1925 had 2n = 22 (18m+2sm+2st) and *Apterostigma andense* Lattke, 1997 had n = 11 (4m+3sm+4st) (Fig. 1). The technique used was not successful to physically map the 18S rDNA genes in *Apterostigma* species, irrespective of the number of repeats or the quality of the metaphases. Regarding the microsatellite (GA)_n_ distribution, the chromosomes of *Apterostigma tropicoxa* had a dispersed pattern, except for the short arms of some chromosomes (Fig. 3c). In *Apterostigma jubatum* the signals were dispersed among both arms of all chromosomes (Fig. 3d), whereas in *Apterostigma andense*, it was also dispersed, with stronger accumulation at the terminal regions of some chromosomes (Fig. 3e).

## Discussion

All the *Cyphomyrmex* colonies examined in this study cultivate yeast and therefore were included in the rimosus group (Kempf 1966; Hanisch et al. 2022). Morphological analysis suggests that *Cyphomyrmex rimosus* may represent a species complex (Snelling and Longino 1992; Albuquerque 2014), which is corroborated by previous cytogenetic analysis (Teixeira et al. 2023). The numerical and morphological differences between the karyotypes of isolated populations of *C. transversus*, which has a chromosomal variation of 2n = 18–42, reinforces the hypothesis of the existing species complex (Mariano et al. 2019; Aguiar et al. 2020; Cardoso and Cristiano 2021; Teixeira et al. 2021b; Teixeira et al. 2023). The discussions focusing only on the morphological variation of rimosus group do not yet support a species complex hypothesis for this taxon (Snelling and Longino 1992; Albuquerque 2014). However, the large differences in the chromosome numbers of these taxa, which have been considered the same species up to now, indicate that this taxon may include several widely distributed species. The rimosus group requires further comprehensive and integrative studies due to the observed intraspecific variation, which will allow for better identification of taxa (Albuquerque 2014).

Chromosomes of the Amazonian *C. transversus* differ from those of individuals collected in other localities. Although the variation in the chromosome number and morphology of this taxon is high (2n = 18, 24, and 42) (Mariano et al. 2019; Aguiar et al. 2020; Cardoso and Cristiano 2021; Teixeira et al. 2021b), the same number of chromosomes is observed in other Amazonian colonies, collected in French Guiana, which also have 2n = 24 (14m+6sm+4a) chromosomes (Aguiar et al. 2020). In *C. transversus* individuals used in this study, the 18S rDNA gene was mapped in the long arm of the first submetacentric pair. In *C. transversus* of Amazonian population from French Guiana previously studied by Aguiar et al. (2020), a remarkable secondary constriction on the long arm of the largest submetacentric chromosome was detected. In addition, a population from Atlantic Forest, with 2n = 18 chromosomes, had its rDNA genes physically mapped to the short arm of the second pair of metacentric chromosomes (Teixeira et al. 2021b). Assuming that chromosome fusions or fissions resulted in the variation in the chromosome number between these two karyotypes, the position of the rDNA genes further suggests that chromosome inversions also play a significant role in the karyotype evolution of this lineage.

In contrast, the karyotype observed in the *C. laevigatus* colony used in this study with n = 7, with all metacentric chromosomes, and rDNA genes mapped to the short arm of the fifth chromosome, is similar to the diploid karyotype previously described for this population (Damasceno et al. 2024). All *Cyphomyrmex* species showed a single pair of chromosomes bearing rDNA genes, pattern observed in most ant species, which is considered an ancestral characteristic within this group (Teixeira et al. 2021a; Damasceno et al. 2024), as well as in insects in general (Gokhman and Kuznetsova 2024). When comparing the populations of *C. transversus* and *C. laevigatus* from Oiapoque, as well as the *C. rimosus* and *C. transversus* from Atlantic Forest, it was observed that the rDNA genes were absent from the terminal regions of the chromosomes, thus supporting the hypothesis proposed by Teixeira et al. (2021a) and Damasceno et al. (2024). It postulates that the terminal positioning of rDNA clusters on ant chromosomes is closely linked to their ability to disperse throughout the genome through ectopic recombination between these genes and terminal repetitive sequences during meiosis (Teixeira et al. 2021a; Damasceno et al. 2024).

In *Cyphomyrmex*, rDNA clusters were observed to be located in the intrachromosomal region in all described karyotypes. Although only a few species have been studied, these findings suggest that the increase in the chromosome number has affected the location of these genes on different chromosome pairs among species of the genus; however, the number of rDNA sites remains conserved, according to Teixeira et al. (2021a) and Damasceno et al. (2024). In *Cyphomyrmex*, 18S rDNA clusters have been localized to distinct chromosomal regions: within the pericentromeric regions of submetacentric chromosomes, as observed in *C. transversus*; in the pericentromeric region of the long arm of a metacentric chromosome, as reported for *C. rimosus* (Teixeira et al. 2023); and in the short arm of metacentric chromosomes, as in *C. laevigatus*. Menezes et al. (2021) proposed that the accumulation of repetitive elements such as 45S rDNA genes in pericentromeric regions may facilitate chromosomal reorganization. In *Cyphomyrmex* species, the expansion or reduction of these repeats is likely associated with alterations in chromosome morphology, which are defined by the arm ratio (Levan et al. 1964). In their comprehensive review of ribosomal DNA (rDNA) data across the class Insecta, Gokhman and Kuznetsova (2024) argue that the hypotheses proposed by Teixeira et al. (2021a) and Menezes et al. (2021) are complementary. The first hypothesis explains broader patterns observed in many insect groups, in which closely related species with similar chromosome numbers may show differences concerning the rDNA clusters (number, size and location of rDNA clusters). The second hypothesis imply that chromosome fissions may facilitate the relocation of these genes to subterminal and terminal regions, thereby promoting recombination during meiosis. Furthermore, the dispersal of rDNA clusters may be associated with transposition mediated by transposable elements inserted into their intergenic spacers. These additional copies may subsequently undergo pseudogenization, as reviewed in Gokhman and Kuznetsova (2024).

*C. transversus* and *C. rimosus* from the Atlantic Forest have already had their microsatellite (GA)_n_ clusters mapped (Teixeira et al. 2022, 2023), and the same pattern of these microsatellites could be observed in the specimens from Amazonian colonies of *C. transversus*, dispersed throughout the chromosomes except for the centromeric/pericentromeric regions. Microsatellite (GA)_n_ clusters had not yet been physically mapped in *C. laevigatus*, which also presented a dispersed pattern on both chromosome arms of all chromosomes. The (GA)_n_ microsatellite has been studied in ten genera of fungus-growing ants, and in most of the studied species, this microsatellite is frequently found dispersed in the euchromatin (Teixeira et al. 2022, Micolino et al. 2022). Only *Mycocepurus goeldii* (Forel, 1893) and *Sericomyrmex* sp. showed additional clustering of (GA)_n_ in some regions (Teixeira et al. 2022).

Individuals of *A. tropicoxa* had the highest chromosome number for the genus, with 2n = 54 chromosomes. Previous data had showed that the highest chromosome number ever described for the genus was observed in *Apterostigma* sp. (pilosum complex) collected in French Guiana, with 2n = 46 (Barros et al. 2021b). Concerning the cytogenetic data for *Apterostigma* species, the individuals of *Apterostigma jubatum*, which have a diploid chromosome number of 22, and *A. andense*, which have a haploid chromosome number of 11, are identical in terms of their diploid/haploid chromosome numbers. However, the morphology of their chromosomes differs significantly. In *Apterostigma jubatum* a high proportion of metacentric chromosomes (18m+2sm+2st) is observed, in relation to *A. andense* with (4m+3sm+4st) (Fig. 1).

In *Apterostigma*, karyotypes with both high and low chromosome numbers are characterized by a predominance of metacentric and submetacentric chromosomes. This pattern differs from karyotypes with high chromosome numbers and a high proportion of subtelocentric chromosomes, which are expected after chromosome fissions according to the Minimum Interaction Theory (Imai et al. 1988, 1994). Following fission events, heterochromatin expansion may occur to stabilize telomeric regions, a process that may have taken place in *Apterostigma*. In addition, the differences in the karyotype of *Apterostigma jubatum* and *Apterostigma andense* suggest that pericentric inversions may have played a significant role in the karyotype evolution in *Apterostigma*.

The species *Apterostigma tropicoxa* recorded from Peru and Brazil, the states of Amazonas, Minas Gerais, and Pará (Lattke 1997; Albuquerque et al. 2021; Santos et al. 2006). *Apterostigma jubatum* was previously recorded in a region ranging from Bolivia to Costa Rica, and also in some Brazilian states (Lattke 1997; Mera-Rodríguez et al. 2020). Recently, the species was collected in two municipalities in the state of Pará (Albuquerque et al. 2021). This is the first record of the species *A. tropicoxa* and *A. jubatum* in the state of Amapá, and also the first study with cytogenetic data. Regarding *A. andense*, the only records of this species were from Peru and Venezuela, in a genus review, the species name itself refers to the Andes Mountains, where the first individuals of the species were collected (Lattke 1997). Thus, this is the first record of the species in Brazil, and with this, we can assume that its distribution may be wider than previously known, with potential distribution throughout the Guiana Shield.

*Apterostigma tropicoxa* showed lower concentration of microsatellite (GA)_n_ in the short arms of several submetacentric and subtelocentric chromosomes. This underscores the significance of centric fission events followed by tandem heterochromatin growth, as proposed by the Minimal Interaction Theory (Imai et al. 1988). The patterns observed in the distribution of (GA)_n_ in *Apterostigma tropicoxa* align with those observed in the individuals from Atlantic Forest colonies of *A. madidiense*, in which this microsatellite was absent from the short arm of some submetacentric chromosomes (Teixeira et al. 2022). The microsatellite (GA)_n_ pattern of *Apterostigma jubatum* was not informative since it was scattered throughout all the chromosomes. In *Apterostigma andense*, high concentrations of this microsatellite are observed at the ends of most chromosomes, the species presents a karyotype characterized by a smaller number of chromosomes, including all metacentrics.

We have obtained numerous high-quality metaphases for all studied *Apterostigma* species but were unable to successfully map 18S rDNA genes on the chromosomes of this genus. The probe derived from genomic DNA of *Camponotus rufipes* enabled the physical mapping of 18S ribosomal genes across several ant species belonging to other distinct genera (Teixeira et al. 2021a; Damasceno et al. 2024). Furthermore, we use a positive control with metaphases of *C. transversus* and *C. laevigatus*, from which we obtained 18S ribosomal gene labeling results in this study. Developing a labeled probe from *Apterostigma* represents an important future direction, however, the causes underlying the observed negative signals are still unknown.

## Conclusion

Our results expand the existing cytogenetic data on fungus-growing ants in northern Amazon with the new information on the genera *Cyphomyrmex* and *Apterostigma*. The taxon *C. transversus* seems to be a species complex with highly diverse chromosome patterns. However, the results presented here support the hypothesis that only a single cytotype of this species is present in the Amazonian Guiana Shield. The cytogenetic data showed that *C. laevigatus* also exhibits taxonomic stability in this region of the Amazon. Furthermore, the physical mapping of the (GA)_n_ microsatellite aligns with those observed in *C. transversus* and *C. rimosus*, despite the subtle differences in their karyotypes.

Cytogenetic studies of Amazonian *Apterostigma* species revealed that the karyotypic diversity within the genus exceeds previous observations, emphasizing the need for extensive sampling in this region and cautious analysis of cytogenetic data on these species. *Apterostigma tropicoxa* has the largest number of chromosomes ever described for the genus, with variations in chromosome morphology in relation to *A. madidiense* and *Apterostigma* sp. *pilosum* complex. This is the first cytogenetic study on species of the genus *Apterostigma* in Brazillian Amazon, and it includes the first cytogenetic descriptions of the species *A. jubatum*, *A. tropicoxa*, and *A. andense*.

The inability to physically map the rDNA genes for the three *Apterostigma* species suggests a necessary methodological adjustment and raises questions about their ribosomal sequences and distribution patterns across the chromosome sets. The observed patterns in the distribution of microsatellites (GA)_n_ among their karyotypes, when compared to the proportion of metacentric chromosomes, support the applicability of the Minimal Interaction Theory to these ants. However, the high proportion of metacentric chromosomes in the karyotype of *Apterostigma tropicoxa*, which has more chromosomes, demands further discussion. Future investigations involving other microsatellite probes or different markers will shed light on the primary mechanisms driving the karyotypic evolution of ants from these two genera.

## Supplementary materials

Supplementary material 1 Cytogenetic data on chromosome number and karyotypic formula of available for species of *Cyphomyrmex* and *Apterostigma* in the literature and present study Data type pdf https://binary.pensoft.net/file/1569043This dataset is made available under the Open Database License (http://opendatacommons.org/licenses/odbl/1.0/). The Open Database License (ODbL) is a license agreement intended to allow users to freely share, modify, and use this Dataset while maintaining this same freedom for others, provided that the original source and author(s) are credited. Rodrigo Batista Lod
