# Supplementary material for: Cytogenetic analysis of some species of Cyphomyrmex Mayr, 1862 and Apterostigma Mayr, 1865 (Formicidae, Myrmicinae) from the Guiana Shield
Source: Comp Cytogenet. 2026 Mar 25;20:99–113. doi: 10.3897/compcytogen.20.175870 (PMC13044544; doi:10.3897/compcytogen.20.175870)
Supplement: Supplementary material 1 — Cytogenetic data on chromosome number and karyotypic formula of available for species of Cyphomyrmex and Apterostigma in the literature and present study [file comparative_cytogenetics-20-099_article-175870__-s001.pdf]

Supplementary table: Cytogenetic data on chromosome number (2n=diploid, n=haploid) and karyotypic formula available for species of *Cyphomyrmex* and *Apterostigma* in the literature and present study. The terminology used for karyotype formulae is in accordance with the published data.

Species	Locality	2n/(n)	Karyotype formula	References
Cyphomyrmex cornutus	French Guiana	22	10M+12SM	Mariano et al. (2011)
Cyphomyrmex costatus	Panamá	20	20M	Murakami et al. (1998)
Cyphomyrmex rimosus	Panamá	32	28M+4A	Murakami et al. (1998)
Cyphomyrmex rimosus [a,b]	Minas Gerais, Brazil	22	18m+4sm	Teixeira et al. (2023)
Cyphomyrmex transversus	French Guiana	24/(12)	14m+6sm+4a	Aguiar et al. (2020)
Cyphomyrmex transversus	São Paulo, Brazil	42	42A	Mariano et al. (2019)
Cyphomyrmex transversus [a,b]	Minas Gerais, Brazil	18	10m+2sm+6a	Teixeira et al. (2021b; 2022)
Cyphomyrmex transversus	Rio de Janeiro, Brazil	42	28m+14sm	Cardoso and Cristiano (2021)
Cyphomyrmex transversus	Amapá, Brazil	24	18m+6sm	This study
Cyphomyrmex laevigatus [a]	Amapá, Brazil	14	14m	Damasceno et al. (2024)
Cyphomyrmex laevigatus [a,b]	Amapá, Brazil	(7)	7m	This study
Cyphomyrmex sp.	Minas Gerais, Brazil	32	14M+18A	Mariano et al. (2019)
Apterostigma andense [b]	Amapá, Brazil	(11)	4m+3sm+4st	This study

Apterostigma jubatum [b]	Amapá, Brazil	22	18m+2sm+2st	This study
Apterostigma madidiense [b]	Minas Gerais, Brazil	(23)	(7m+10sm+5st+1a)	Barros et al. (2013), Teixeira et al. (2022)
Apterostigma madidiense	Minas Gerais, Brazil	24	24m	Cardoso and Cristiano (2021)
Apterostigma mayri	Panamá	24	24M	Murakami et al. (1998)
Apterostigma steigeri	Minas Gerais, Brazil	22	20m+2sm	Barros et al. (2013)
Apterostigma tropicoxa	Amapá, Brazil	54	20m+30sm+4st	This study
Apterostigma sp. *pilosum* complex	French Guiana	46	6m+18sm+16st+6a	Barros et al. (2021b)
Apterostigma sp.	Panamá	24	24M	Murakami et al. (1998)
Apterostigma sp.	Minas Gerais, Brazil	20	6M+12SM+2A	Fadini and Pompolo (1996)
Apterostigma sp.	French Guiana	32	14M+6SM+10ST+2T	Mariano et al. (2011)

a - Physical mapping of 18S rDNA available.
b - Physical mapping of $(GA)_n$ clusters available.
